# Supplementary figures and images for: Mutations in fibulin-1 and collagen IV suppress the short healthspan of mig-17/ADAMTS mutants in Caenorhabditis elegans
Source: PLoS One. 2024 Jul 9;19(7):e0305396. doi: 10.1371/journal.pone.0305396 (PMC11232982; doi:10.1371/journal.pone.0305396)

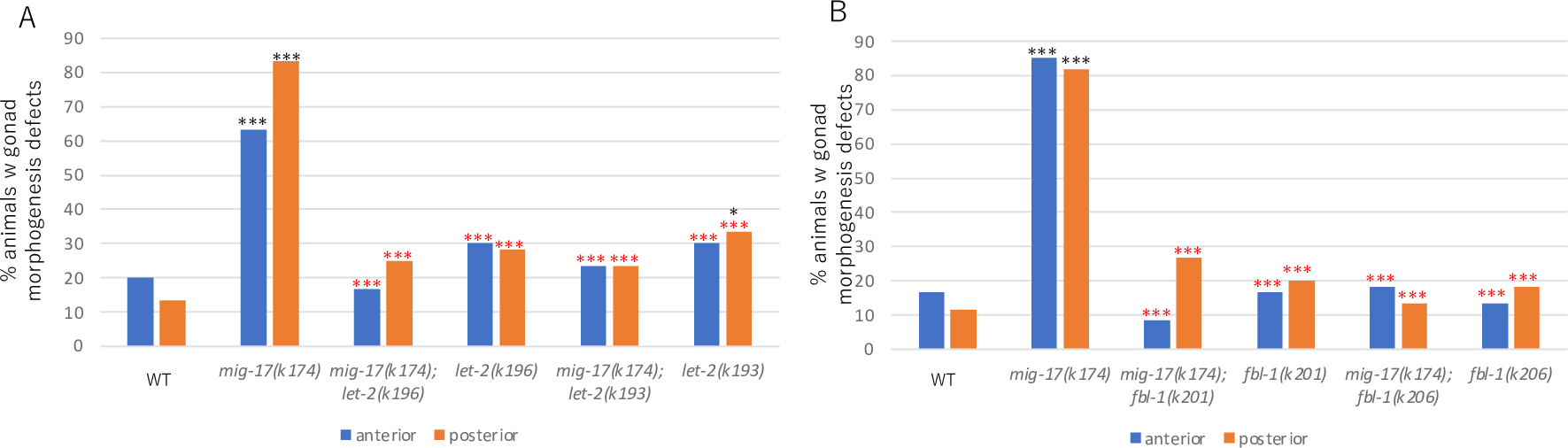

Supplement: S1 Fig — Percentage of DTC migration defects. Blue and orange bars represent defects in anterior and posterior gonad arms, respectively. Black and red asterisks indicate p-values for Fisher’s exact test against WT and mig-17(k174), respectively p-values for Fisher’s exact test are indicated: ***p < 0.005, *p < 0.05. (TIF) [file pone.0305396.s001.tif]
