# Supplementary material for: Mutations in fibulin-1 and collagen IV suppress the short healthspan of mig-17/ADAMTS mutants in Caenorhabditis elegans
Source: PLoS One. 2024 Jul 9;19(7):e0305396. doi: 10.1371/journal.pone.0305396 (PMC11232982; doi:10.1371/journal.pone.0305396)
Supplement: Attachment — Submitted filename: Response to the reviewer 1st.docx [file pone.0305396.s002.docx]

Dear Editor,

Thank you for serving as the editor of our manuscript entitled " Mutations in fibulin-1 and collagen IV suppress the short healthspan of *mig-17/ADAMTS* mutants in *Caenorhabditis elegans* “ by Yukimasa Shibata, Yijing Huang, Moeka Yoshida, Kiyoji Nishiwaki. We have revised our manuscript according to the reviewer’s comments. We think this manuscript is an improved representation of our work, and hope it can be readily accepted for publication in *PLos One*.

Sincerely,

Followings are responses to editor’s and reviewer’s comment.

*Comments to the Author*

*Reviewer #1: This paper from a leader in the C. elegans organogenesis field describes how matrix proteins contribute to organismal aging. In addition to its role in DTC migration, the metalloprotease MIG-17/ADAMTS is important for healthy aging in the worm. Two other matrix proteins, LET-2/collagen and FBL-1/fibulin that interact genetically with MIG-17 in DTC migration also suppress age-related phenotypes in the mig-17 mutants in an allele-specific manner. I think this will be of interest to those interested in matrix and aging.*

*Recommendations:*

*1. Make it more clear in the abstract that let-2 and fbl-1 differentially affect the lifespan of mig-17 mutants, and that mig-17 shows age related phenotypes (say what these are) but not short lifespan per se. It might be more correct to say that mig-17 shows altered healthspan, not altered lifespan.*

We added underlined phrase in the abstract.

Page 3, line 11

“Interestingly, *fbl-1(k206)*, but not *fbl-1(k201)* or *let-2* alleles, exhibited an extended lifespan compared to the wild type when combined with *mig-17*.”

Page 3, line 14

“In addition to the control of DTC migration, MIG-17 also plays a role in healthspan, but not in lifespan”

*2. Provide a bit more information in the introduction about the roles of basement membrane in aging. What is known about this? (e.g. skin aging and collagen). This will make the article more accessible to a broader audience. Also provide a brief general description of the mig-17 age-dependent phenotypes.*

We added following sentences to describe the roles of basement membrane in aging.

Page 4, line2

“The BMs are complex structures composed of multiple types of molecules. To maintain their integrity, it is necessary to appropriately remove damaged components and incorporate new ones, ensuring proper turnover. However, as animals age, the metabolism of BMs slows down, leading to the accumulation of damage [1]. Collagen levels decrease with age, while spontaneous cross-links increase. Yet, the anti-aging effects of BMs remain poorly understood. In the skin, BM collagen is essential for stem cell maintenance through hemidesmosomes [2]. Loss of collagen is known to impair cell competition, thus contributing to aging. However, the relationship between BMs and the suppression of aging beyond skin stem cells remains unknown.”

Accordingly, we added two references

1.	Ewald CY. The Matrisome during Aging and Longevity: A Systems-Level Approach toward Defining Matreotypes Promoting Healthy Aging. Gerontology. 2020;66(3):266-74. Epub 20191213. doi: 10.1159/000504295. PubMed PMID: 31838471; PubMed Central PMCID: PMCPMC7214094.

2.	Liu N, Matsumura H, Kato T, Ichinose S, Takada A, Namiki T, et al. Stem cell competition orchestrates skin homeostasis and ageing. Nature. 2019;568(7752):344-50. Epub 20190403. doi: 10.1038/s41586-019-1085-7. PubMed PMID: 30944469.

To describe the *mig-17* age-dependent phenotypes, we modified the description as follows.

Page 5, 3rd line from the bottom

“*mig-17* mutants exhibit an earlier decline in periodic behavior including the defecation cycle and pumping rate, and motility rate, as well as earlier shortening of body length compared to the wild type*”*

*3. Methods: Please provide more detail about the imaging (DIC, I assume?).*

We added underlined phrase.

Page 6, 5th line from the bottom

“Gonad migration phenotypes were scored using a DIC images of Nomarski microscope (Axioplan 2; Zeiss).”

*4. Statistics: Please also include a section describing the statistical methods. Each figure legend should more clearly describe the statistical methods used (just saying t-test is not sufficiently descriptive). Multiple comparisons corrections should be applied.*

Thank you for your suggestion. We performed multiple comparisons corrections in Fig. 3, 4, 5 using one-way ANOVA and Tukey’s multiple comparisons.

Accordingly, we changed figure legends as follows.

Legend of figure 3-5

We removed “for t-test” from following sentence “p-values for t-test against WT is indicate at the bottom of plots.”

We replaced“: *** *p*<0.005, ** *p*<0.01, **p*<0.05.” to “One-way ANOVA with Tukey’s multiple comparisons:, **p*≤ 0.05, ***p*≤ 0.01.”

We added statistics section in Materials and Methods.

Page 8, line 6

“**Statistics**

Growth rates were analyzed using Fisher's exact test to compare adults and larvae.

One-way analysis of variance (ANOVA) was conducted using Excel to compare defecation cycle, pumping rate, and body length. If a significant difference was found by ANOVA, Tukey’s multiple comparison test was performed using Excel statistics.

The lifespan was analyzed using a log-rank test in R.”

Accordingly, we changed result and discussion as follows.

Page 10, 4th line from the bottom

“*fbl-1(k206)* alleles showed significantly longer defecation cycles compared to the wild type after day 5 and *fbl-1(k201)* showed a trend toward suppression of the *mig-17* defecation decline at day 5, although it was not statistically significant (t-test: *p*=0.069)”

Page 11, line 4

“The pumping rates of *mig-17* adults were much slower than those of wild type at day 1 and day 9, suggesting short healthspan of *mig-17* animals (Fig. 4C, D) [11]. Interestingly, *let-2(k196)*, but not *let-2(k193)*, showed a trend toward suppression of the *mig-17* pumping rate decline at day 9 (Fig. 4A, B), although it was not statistically significant. The pumping rate of *mig-17(k174)* and *fbl-1(k206); mig-17(k174)*, but not that of *fbl-1(k201);* *mig- 17(k174)*, showed significant differences from that of wild-type animals at day 9 adult. (Fig. 4C, D).”

Page 11, 6th line from the bottom

“The body length of day 1 adults was longer in *mig-17; let-2(k196)* and *mig-17; let-2(k193)* double mutants compared to the wild type or *mig-17* mutants. At the day3 adult, the body length of *mig-17(k174)* and *let-2(k193) mig-17(k174)*, but not that of *let-2(k196)* *mig- 17(k174)*, showed significant differences from those of wild-type animals”

Page 14, line 8

“However, *let-2(k196)* suppressed the early prolongation of *mig-17* defecation cycle associated with aging.”

We also changed Table1 as follows.

Defecation cycle: *mig-17(k174) fbl-1(k201)* and *fbl-1(k201)* became blank.

Pumping rate: *mig-17(k174) let-2(k193), let-2(k196)*, and *let-2(k193)* became blank.

Body length (represent day3 data): *mig-17(k174) fbl-1(k206)* and *fbl-1(k206)* became short.

*5. Include the supplementary diagram showing the position of the alleles on the proteins in the main text. Include MIG-17 too, and show that the allele studied is a null. This should be figure 1. Also a diagram of the worm and/or a photo showing where these proteins are localized might help.*

We added Figure 1, which includes a diagram showing the position of the alleles on the MIG-17, LET-2, and FBL-1 proteins. Additionally, we included a diagram indicating the localization of these proteins on BMs.

Accordingly, we added legend of Figure 1, as follows.

“Figure 1: Schematic diagrams of MIG-17, FBL-1C, and LET-2A.

(A) The mutation sites of mig-17(k174), let-2(k193, k196), and fbl-1(k201, k206) are indicated. (B) A diagram showing the localization of MIG-17, LET-2, and FBL-1. These proteins are components of BMs.

”

And we removed the description of alleles of FBL-1 and LET-2 from Sup Figure1.

Accordingly, we changed Fig. 1-5 to Fig. 2-6 and removed Sup Figure1A.

*6. Fig. 4 show all data as in Fig. 3. Not clear why there are asterisks above and below the columns. Effects seem very minor – check for multiple comparisons correction in statistics.*

Thank you for your suggestion. We represented Fig. 5(ex-Fig. 4) as box and dot plot.

Accordingly, we changed first sentence of legend as follows.

“Box and dot plots indicate the body length.”

*7. Fig. S1 % abnormal animals – abnormal in what way? Gonad morphogenesis defects would be more descriptive.*

We changed description of y axis to “% animals w gonad morphogenesis defects”

Minor:

*p.5 Change ‘In addition to its plays a role in gonadogenesis, MIG-17 also function in regulating healthy aging’ to ‘In addition to its role in in gonadogenesis, MIG-17 also functions in regulating healthy aging’.*

We followed reviewer’s suggestion.

*p. 6 ‘young adult animals were collected as day 1 adults’ and ‘number of pumps during 30 seconds’ ‘Average of three independent measurements were calculated for each individual’*

We followed reviewer’s suggestion.

*p. 7 Add a period after ‘punctuated by molting’.*

We followed reviewer’s suggestion.

*p.9 ‘Additionally we investigated changes in body length during aging in this study’. This sentence seems out of place. Recommend delete and just merge the previous sentence in with the next paragraph.*

We followed reviewer’s suggestion.

*p. 9 last sentence Delete ‘were observed to’. It is fine to just say ‘The pumping rates decreased in day 9 adults’. Better would be to provide the quantification in the text. E.g decreased by 20% or whatever it is.*

We changed to “The pumping rates decreased by 50% in day 9 adults.” (Page 10, 1^st^ line)

*p.15 ‘Fibulin-1 is also known to regulate…’*

We followed reviewer’s suggestion.

*Fig. 1 Change ‘hatch’ to ‘hatching’ . T-tests not the appropriate stat for comparing percentages. Recommend using Chi-squared or Fisher’s exact.*

We followed reviewer’s suggestion.

Accordingly, we changed figure legend as follows.

“Black and red asterisks on the top of bar graph indicate *p*-values for Fisher’s exact test against WT and *mig-17(k174),* respectively”

In addition, we performed Fisher’s exact test to Supplementary data that analyzed suppression of the gonad migration defect of *mig-17*.

*All figures: X axis fonts are very tiny. Turning these to an angle might help.*

We followed reviewer’s suggestion.

*Fig. 3 Y axis: Number of pumps in 30 sec*

We followed reviewer’s suggestion.

Fig. 5 fonts way too tiny

We followed reviewer’s suggestion.

*Fig. 6 should be Table 1.*

We followed reviewer’s suggestion.

*Reviewer #2: This manuscript describes the genetic interaction between mig-17 and fbl-1 or let-2 viable alleles. The authors argue that allele-specific suppression of age-related phenotypes of the mig-17 null allele is present. This paper is an extension of their work published previously. This manuscript only displays behavioral analyses without cell biological data. However, their studies can provide important insight. The remodeling of the matrix may affect an animal's age-related markers.*

*While it contains exciting studies, the manuscript requires improvements to be published in PLOS One.*

*1. Introduction. The author should summarize how cell-matrix interaction links to age-related phenotypes, including examples from other systems.*

Please see response to reviwer1 point 2.

*2. Materials and Methods. The authors must include details of procedures (p.6). ‘Body length was measured using ImageJ software’; I suggest adding more detail. “ … the growth was assessed on the stage of vulva development …” also needs more detail.*

We added following sentences to explain details of procedures.

Page 7, line 8

“To measure body length, we traced a line along the body using ImageJ and then measured its length.”

Page 7, line 2

“A simple invagination occurs in early L4, followed by a Christmas tree-like invagination in mid-L4, and the invagination becomes mostly closed in late L4.”

*3. Age-related phenotype suppression (Figure 5, difficult to read). I noticed that the median life span of mig-17 in panels A, B, and C ranged from 18 to 21 days, but the median of mig-17 in panel D appears to be around 16 days. The authors need to clarify the differences. Or, the experiment must be repeated.*

*C. elegans* lifespans vary from experiment to experiment, both for wild type and mutants. This variability is believed to be influenced by multiple factors, including the degree of dryness of the plate and the amount of *E. coli* used as food. Given the difficulty in controlling these conditions, when measuring the lifespan of *C. elegans*, we compare the control and sample using plates seeded with *E. coli* prepared simultaneously. Discrepancies in lifespan may arise if plates are created at different times. The variance in the lifespan of *mig-17* between experiments falls within the normal range. Moreover, in both instances, the results are reproducible, as there are no significant differences observed between the wild type and *mig-17* mutants.

*4. Lifespan phenotypes of mutants. The authors described the reduced lifespan. In general, any mutations can reduce lifespan. Although the genetic study is interesting, it is difficult to convince that such mutations directly affect lifespan.*

We agree with reviewer’s suggestion. Therefore, we added underlined phrase in discussion part.

Page 14, line 6

“Thus, both of these type IV collagen mutations have a negative impact on lifespan, although it is not clear whether these mutations directly affect lifespan.”

*5. Minor comments.*

*a. Some typos need to be corrected. Ex) 'let-2(196)' and 'TGFβ'.*

We corrected these typo.

*b. Graphs are difficult to understand. Figure 5 graph must be drawn using thicker lines.*

We followed reviewer’s suggestion.

*c. Overall, the manuscript needs to be revised to a cohesive format.*

We followed reviewer’s suggestion.
