# Supplementary material for: Mutations in fibulin-1 and collagen IV suppress the short healthspan of mig-17/ADAMTS mutants in Caenorhabditis elegans
Source: PLoS One. 2024 Jul 9;19(7):e0305396. doi: 10.1371/journal.pone.0305396 (PMC11232982; doi:10.1371/journal.pone.0305396)
Supplement: Attachment — Submitted filename: Response to the reviewer 2nd.docx [file pone.0305396.s003.docx]

Dear Editor,

Thank you for serving as the editor of our manuscript entitled " Mutations in fibulin-1 and collagen IV suppress the short healthspan of *mig-17/ADAMTS* mutants in *Caenorhabditis elegans* “ by Yukimasa Shibata, Yijing Huang, Moeka Yoshida, Kiyoji Nishiwaki. We have revised our manuscript according to the reviewer’s comments. We think this manuscript is an improved representation of our work, and hope it can be readily accepted for publication in *PLos One*.

Sincerely,

Followings are responses to editor’s and reviewer’s comment.

*Comments to the Author*

*Reviewer #1: Change the Microscopy section in the materials and methods to:*

*Gonad migration phenotypes were scored using DIC images captured with a Zeiss Axioplan 2 microscope.*

Thank you for your suggestion. We followed reviewer’s comment.

*Under ‘Behavioral Analysis’, recommend edits to make complete sentences: ‘Number of pumps during 30 seconds were counted and the average of three independent measurements was calculated for each individual.*

We followed reviewer’s comment.

*Reviewer #2: 1. Please clarify the purpose of Figure 1. The domain structures were not mentioned in the Introduction. Specifically, Figure 1B should have been mentioned in the text. I suggest considering its deletion.*

We cited Figure 1A, and 1B in Introduction as follows.

Page 5 line2

“MIG-17 is secreted from the body wall muscle cells as a proform and localizes to the BMs of various tissues, including gonad, intestine, body wall muscle, and hypodermis, where it is activated by auto-catalytic removal of its prodomain [5, 6] (Fig. 1A, B).”

Page 5 line7

“Dominant gain-of-function (*gf*) mutations in the BM molecules collagen IV α2 chain and fibulin-1 suppress the gonadal defect of the *mig-17* mutants [8-10] (Fig. 1A). *let-2(gf)* mutations are amino-acid substitutions either in the triple helical domain or in the C-terminal non-collagenous 1 (NC1) domain (Fig. 1A). *fbl-1(gf)* mutations are amino-acid substitutions found in the second EGF-like motif (Fig. 1A).”

*2. Materials and methods. References are needed for the statistical methods used in the study.*

Thank you for your suggestion. We followed reviewer’s comment.

Followings are references.

Shibata Y, Tanaka Y, Sasakura H, Morioka Y, Sassa T, Fujii S, et al. Endogenous chondroitin extends the lifespan and healthspan in C. elegans. Sci Rep. 2024;14(1):4813. Epub 20240227. doi: 10.1038/s41598-024-55417-7. PubMed PMID: 38413743; PubMed Central PMCID: PMCPMC10899230.

Choi W, Ryu SE, Cheon Y, Park YJ, Kim S, Kim E, et al. A single chemosensory GPCR is required for a concentration-dependent behavioral switching in C. elegans. Curr Biol. 2022;32(2):398-411 e4. Epub 20211213. doi: 10.1016/j.cub.2021.11.035. PubMed PMID: 34906353.

*3. Page 9, line 9. “the gain of function mutations in collagen IV..” needs a reference.*

We followed reviewer’s comment.

Followings are references.

Kubota Y, Kuroki R, Nishiwaki K. A fibulin-1 homolog interacts with an ADAM protease that controls cell migration in C. elegans. Curr Biol. 2004;14(22):2011-8. doi: 10.1016/j.cub.2004.10.047. PubMed PMID: 15556863.

Imanishi A, Aoki Y, Kakehi M, Mori S, Takano T, Kubota Y, et al. Genetic interactions among ADAMTS metalloproteases and basement membrane molecules in cell migration in Caenorhabditis elegans. PLoS One. 2020;15(12):e0240571. doi: 10.1371/journal.pone.0240571. PubMed PMID: 33264296; PubMed Central PMCID: PMCPMC7710118.

*4. Page 11, line 7. “.. showed significant differences ..” needs a p-value in parenthesis.*

We added p-value as follows.

Page 11, line 7

“The pumping rate of *mig-17(k174)* (*p*=0.0001) and *fbl-1(k206) mig-17(k174)* (*p*=0.028), but not that of *fbl-1(k201);* *mig-17(k174)* (*p*=0.105), showed significant differences from that of wild-type animals at day 9 adult (Fig. 4C, D).”
